# Bacterial vs viral etiology of fever: A prospective study of a host score for supporting etiologic accuracy of emergency department physicians

**DOI:** 10.1371/journal.pone.0281018

**Published:** 2023-01-30

**Authors:** Meirav Mor, Meital Paz, Lisa Amir, Itzhak Levy, Oded Scheuerman, Gilat Livni, Claire Guetta-Oz, Sivan Yochpaz, Ron Berant, Rama Schwartz, Omer Niv, Dana Singer, Shai Ashkenazi, Yehezkel Waisman

**Affiliations:** 1 Schneider Children’s Medical Center, Petach Tikva, Israel; 2 Tel Aviv University, Tel Aviv, Israel; 3 Carmel Medical Center, Haifa, Israel; 4 Technion Israel Institute of Technology, Ruth and Bruce Rappaport Faculty of Medicine, Haifa, Israel; 5 Dana Dwek Children’s Hospital, Tel Aviv Sourasky Medical Center, Tel Aviv, Israel; 6 Adelson School of Medicine, Ariel University, Ariel, Israel; Shiraz University of Medical Sciences, ISLAMIC REPUBLIC OF IRAN

## Abstract

**Background:**

A host-protein score (BV score) that combines the circulating levels of TNF-related apoptosis-inducing ligand (TRAIL), interferon gamma-induced protein 10 (IP-10) and C-reactive protein (CRP) was developed for distinguishing bacterial from viral infection. This study assessed the potential of the BV score to impact decision making and antibiotic stewardship at the emergency department (ED), by comparing BV score’s performance to physician’s etiological suspicion at patient presentation.

**Methods:**

Rosetta study participants, aged 3 months to 18 years with febrile respiratory tract infection or fever without source, were prospectively recruited in a tertiary care pediatric ED. 465 patients were recruited, 298 met eligibility criteria and 287 were enrolled. ED physician’s etiological suspicion was recorded in a questionnaire. BV score was measured retrospectively with results interpreted as viral, bacterial or equivocal and compared to reference standard etiology, which was adjudicated by three independent experts based on all available data. Experts were blinded to BV scores.

**Results:**

Median age was 1.3 years (interquartile range 1.7), 39.7% females. 196 cases were reference standard viral and 18 cases were reference standard bacterial. BV score attained sensitivity of 88.9% (95% confidence interval: 74.4–100), specificity 92.1% (88.1–96.0), positive predictive value 53.3% (35.5–71.2) and negative predictive value 98.8% (97.1–100). Positive likelihood ratio was 11.18 (6.59–18.97) and negative likelihood ratio was 0.12 (0.03–0.45). The rate of BV equivocal scores was 9.4%. Comparing physician’s suspicion to BV score and to the reference standard, and assuming full adoption, BV score could potentially correct the physician’s diagnosis and reduce error ~2-fold, from 15.9% to 8.2%.

**Conclusions:**

BV score has potential to aid the diagnostic process. Future studies are warranted to assess the impact of real-time BV results on ED practice.

## Introduction

Fever is common in children presenting to the emergency department (ED). A presumptive diagnosis is made following history and physical examination and may, in some cases, be supported by the results of routine laboratory biomarkers such as white blood cell count, absolute neutrophil count and C-reactive protein (CRP). However, these biomarkers are constrained by patient-to-patient variability and thus their utility is limited [1–4]. Other tests are either beyond the scope of ED evaluation (e.g., PCR for respiratory pathogens and serology) or require extended time to results (e.g., cultures) and are thus not available to the ED physician in real-time decision making. The resulting diagnostic uncertainty can lead to sub-optimal patient management, including the precautionary use of antibiotics [5, 6]. There is therefore an unmet need for a rapid and accurate diagnostic test to support the physician in distinguishing between bacterial and viral etiologies, to improve diagnosis and decrease unnecessary antibiotic prescription.

To complement the diagnostic tests available to the physician, a host response score was developed for differentiating between bacterial and viral infection, called the BV score. It is based on computational integration of the circulating levels of three immune proteins: tumor necrosis factor-related apoptosis-inducing ligand (TRAIL), interferon gamma-induced protein-10 (IP-10) and CRP [7]. It has demonstrated high diagnostic accuracy as compared to an expert adjudication reference standard in multiple studies [8–13].

The current prospective study focused on patients aged 3 months to 18 years old presenting to the ED with febrile respiratory tract infection (RTI) or fever without source (FWS). These cases are frequently associated with diagnostic uncertainty [5, 6] and therefore a new tool for differentiating between bacterial and viral infection has considerable potential for clinical impact. Here, BV score’s performance is compared to the physician’s etiological suspicion at ED presentation to estimate this tool’s potential to impact decision making and thereby improve patient care.

## Materials and methods

The Standards for Reporting of Diagnostic Accuracy Studies (STARD) checklist can be found in S1 Table.

The study’s minimal underlying dataset can be found in S1 File.

### Patient population

The prospective study was conducted at the ED of Schneider Children’s Medical Center of Israel, a tertiary care pediatric facility, between May 2016 and April 2018. Children aged 3 months to 18 years old with fever ≥38.0°C documented at least once within a week from their presentation, and with clinical suspicion of RTI or with FWS, or with urinary tract infection (UTI) or with gastroenteritis (GE), were prospectively enrolled. Only children for whom blood testing at the ED was deemed necessary were included in the study.

Exclusion criteria included: 48 hours or more of antibiotic treatment at time of presentation; another episode of febrile infection within 2 weeks of presentation; a proven or suspected HIV or hepatitis C or B virus infection; primary or secondary immune deficiency; active malignancy; post-transplant; moderate-to-severe metabolic disorders and other severe chronic conditions.

Ethical clearance was granted by the Rabin Medical Center Institutional Review Board (0666-15-RMC). Written informed consent was obtained from each patient’s legal guardian. If one of the parents was not present in the ED, informed consent from this parent was obtained by telephone. This study was registered at ClinicalTrials.gov (NCT04254991).

### Study design

After enrollment, blood was drawn per treating physician’s discretion. Additional workup and treatment decisions were taken by the treating physician. A serum sample was collected for study-specific BV score measurements from each participant. Also, a nasal swab sample was collected for multiplex polymerase chain reaction (PCR) detection of common respiratory viruses for the reference standard. The BV score and PCR results were not available during the patient’s ED or hospital stay.

For each patient, a questionnaire was filled by the ED treating physician, after history taking and physical examination. In the questionnaire (shown in S1 Fig), the physicians were asked to state their seniority level (intern/specialist), the suspected clinical syndrome and their tentative etiological suspicion. Additionally, physicians were requested to indicate their degree of diagnostic certainty (e.g., viral+, viral++, or viral +++). They were also asked to indicate if relevant test results were available to them at the time of the questionnaire.

The entire medical record, including demographics, medical history, physical examination, laboratory, microbiology and imaging investigation performed as part of routine care, disease course, follow-up data and study-specific serum and nasal swab results was recorded in a case report form. A research assistant contacted the family within 28 days of discharge to collect additional data, specifically additional diagnoses and antibiotic prescription.

The primary objective of the Rosetta study was to assess the performance of the BV score in children with clinical suspicion of RTI or FWS in comparison to a reference standard based on unanimous adjudication. The secondary objectives were to assess the performance of the BV score using a reference standard based on majority adjudication, and to compare the physician’s etiological suspicion to the BV score and unanimous reference standard.

### Reference standard based on expert adjudication

Given the absence of a gold standard for determining bacterial versus viral etiology in real-life patients, the reference standard for determining the etiology of the patients’ disease was generated based on expert panel adjudication in line with the National Health Service (NHS) Health Technology Assessment Guidelines for Evaluation of Diagnostic Tests [14] and previous studies [7–13]. Three pediatricians, each with more than 7 years of experience, independently assessed the case report form for each patient (including clinical, laboratory, microbiological, radiological and follow up data), and assigned one of the following classifications: bacterial (this included both pure bacterial and mixed bacterial-viral infections), viral, non-infectious, or indeterminate. Experts were blinded to their peers’ classifications and to BV score result and were not provided any structured guidelines.

"Bacterial" and "viral" reference standard diagnoses required all the experts to give the same classification. "Suspected bacterial" and "suspected viral" diagnoses were when the majority of experts adjudicated the same classification. "Indeterminate" was when the majority of experts gave this classification or when there was no majority. Indeterminate cases were not included in performance calculations. The reference standard diagnosis matrix is given in S2 Table.

### Index test (BV score)

BV score is based on the computational combination of circulating levels of TRAIL, CRP and IP-10. The serum concentrations of the three host proteins were measured and computationally integrated into a score indicative of bacterial versus viral infection that ranges from 0 to 100 using ImmunoXpert™ (MeMed, Israel). The algorithm for integrating the serum levels of the three proteins was derived based on data from over a thousand patients [7] and is identical to the algorithm used in previous validation studies [7–10]. For more details regarding the algorithm based on logistic regression, see [7]. Cutoffs were validated in previous studies [7–10] and are based on manufacturer’s instructions for use, i.e., score<35 indicates viral infection (or other non-bacterial etiology); score>65 indicates bacterial infection (including co-infection); and 35≤score≤ 65 is considered equivocal. Equivocal scores do not provide etiological information and the physician is advised to employ other patient data in their decision making.

### Laboratory procedures

Blood drawn for study measurements was fractionated within two hours and stored at -20°C. CRP was measured using Cobas c 501 (Roche, Switzerland). TRAIL and IP-10 were measured using ImmunoXpert™ (MeMed, Israel). Of note, ImmunoXpert™ is no longer available; (the BV score can be measured within 15 minutes from serum using a point-of-need immunoassay platform called MeMed Key® and the MeMed BV® test cartridge (MeMed, Israel; CE-IVD and FDA cleared)). The scores generated by ImmunoXpert and running MeMed BV on MeMed Key have been established as comparable [15].

Nasal swab samples (COPAN UTM system, COPAN Diagnostics, California, USA) were stored at 2–8°C for up to 24 hours and then transferred into storage at -20°C. Samples were subjected to Allplex™ Respiratory Panel 1, 2, and 3 at Hy Laboratories Ltd. (Hylabs, Israel).

### Statistical analysis

The diagnostic accuracy of the BV score was calculated in comparison to the reference standard, with bacterial considered “positive”. Sensitivity was defined as the number of patients that received a bacterial diagnosis and a bacterial BV score (score>65) = true positive, divided by the number of patients that received a bacterial diagnosis = (true positive + false negative). Specificity was symmetrically defined as the number of patients that received a viral diagnosis and a viral BV score (score<35) = true negative, divided by the number of patients that received a viral diagnosis = (true negative + false positive). Positive predictive value (PPV) was defined as the number of patients that received a bacterial diagnosis and a bacterial BV score (score>65) = true positive, divided by the number of patients that received a bacterial BV score = (true positive + false positive). Negative predictive value (NPV) was symmetrically defined as the number of patients that received a viral diagnosis and a viral BV score (score<35) = true negative, divided by the number of patients that received a viral BV score = (true negative + false negative). Positive and negative likelihood ratios were defined as sensitivity/(1-specificity) and (1-sensitivity)/specificity, respectively. Cases with equivocal BV scores were not included in the performance calculation and are reported as a rate. Fisher’s exact test was used for comparing proportions.

### Comparing the physician’s clinical suspicion to the BV score (diagnostic accuracy rate)

The following extrapolations were performed to estimate the physician’s practice if the BV score was available: when BV score was equivocal (35≤score≤65), the physician’s original label was employed; when BV score was bacterial or viral (>65 or <35, respectively), BV score was employed (assuming full adoption of BV score by the physician). The accuracy rate was calculated in comparison to the bacterial and viral reference standard diagnoses.

## Results

### Patient characterization

**Fig 1:**
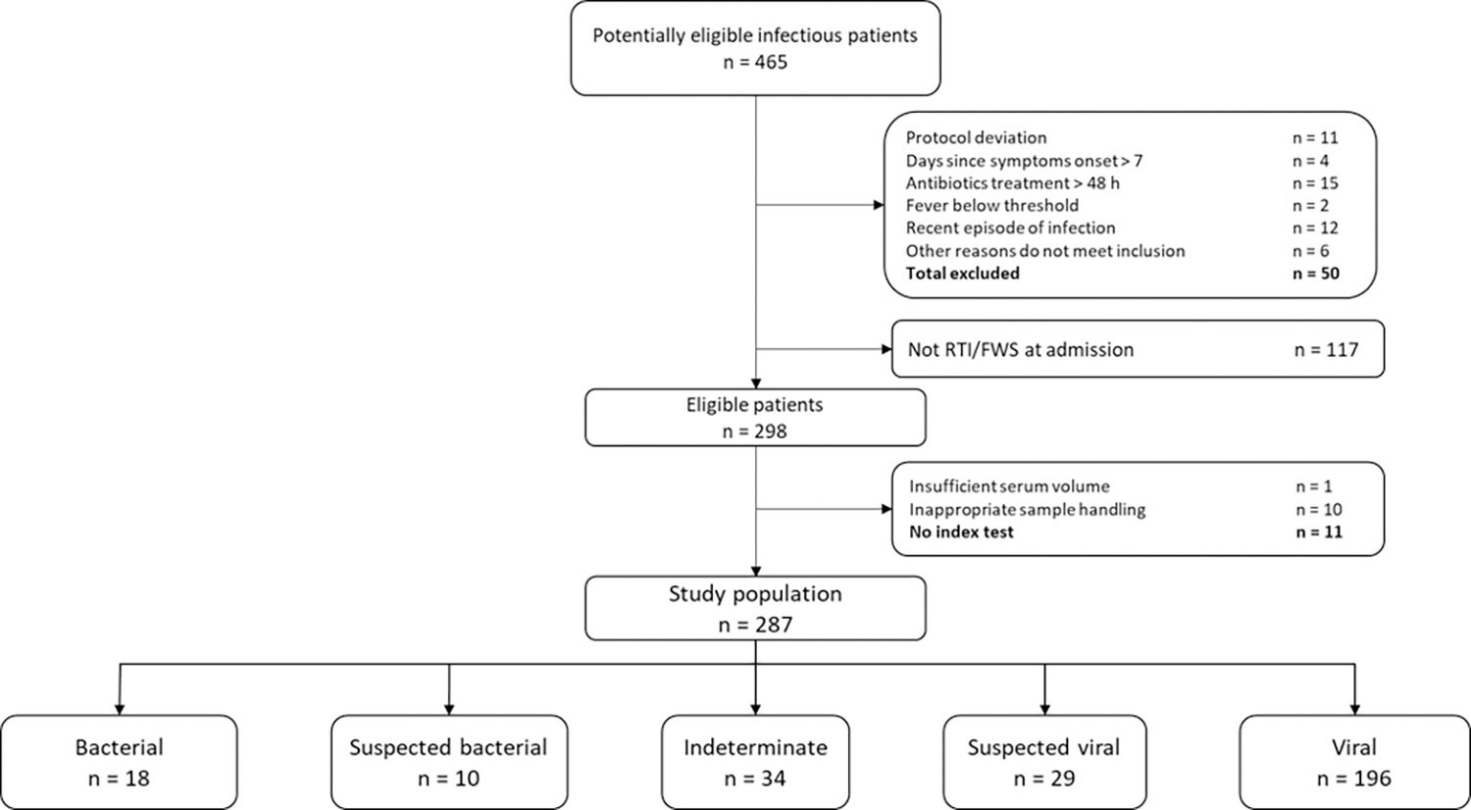
Patient enrollment flow. Summary of patients enrolled in the study. Primary (bacterial/viral) cohort (n = 214) includes reference standard bacterial (n = 18) and reference standard viral (n = 196). Secondary (bacterial/viral/suspected) cohort (n = 253) includes the reference standard bacterial/viral (n = 214) as well as the reference standard suspected bacterial (n = 10) and reference standard suspected viral (n = 29). RTI, respiratory tract infection and FWS, fever without source were based on the suspected clinical syndrome as recorded by the physician in the questionnaire.

Out of 465 potentially eligible subjects, 167 did not meet eligibility criteria and 11 had samples with insufficient volume for measuring BV score; the resulting study population was 287 children (Fig 1). The primary analysis cohort (n = 214) included 196 patients with reference standard viral diagnoses and 18 patients with reference standard bacterial diagnoses. The secondary analysis cohort (n = 253) included, in addition, 29 patients with reference standard suspected viral diagnoses and 10 patients with reference standard suspected bacterial diagnoses. There were 34 cases that could not be assigned a reference standard etiology and were classified as indeterminate.

The mean age of the study population was 1.3 years (interquartile range 1.7). 44 children (15.3%) were admitted (Table 1).

**Table 1:** Demographics of study population (n = 287).

	Study population (n = 287)
**Demographics**	
Age (years)—median (IQR)	1.3 (1.7)
Gender (Female)—n (%)	114 (39.7%)
**Signs and Symptoms**	
Maximal temperature (Celsius)—mean (SD)	39.6 (0.7)
Time from symptom onset (days)—median (IQR)	3.0 (4.0)
**Treatment**	
Received antibiotics in the community^a^ - n (%)	26 (40.6%)
Received antibiotics after admission—n (%)	111 (38.8%)
**Hospitalization**	
Hospitalization rate—n (%)	44 (15.3%)
Hospitalization duration (days)^b^ - median (IQR)	4.5 (4.0)
**Discharge diagnosis**	
LRTI—n (%)	64 (22.3%)
URTI—n (%)	79 (27.5%)
FWS—n (%)	24 (8.4%)
Unspecified viral infection—n (%)	94 (32.8%)
Other^c^ - n (%)	26 (9.1%)

^a^ Among patients for whom information was available (n = 64)

^b^ Among hospitalized patients (n = 44)

^c^ Includes suspected occult bacteremia (n = 8); meningitis (n = 4); gastroenteritis (n = 4); transient synovitis (n = 2); acquired hemolytic anemia (n = 1); convulsions (n = 1); meningoencephalitis (n = 1); non-infectious (n = 1); peritonsillar abscess (n = 1); pyelonephritis (n = 1); unknown (n = 1); UTI (n = 1).

IQR, interquartile range; FWS, fever without source; LRTI, lower respiratory tract infection; URTI, upper respiratory tract infection.

For the 18 patients with bacterial infection in the primary analysis, the discharge diagnoses were as follows: 10 pneumonia cases, 1 acute tonsillitis, 1 acute otitis media (AOM), 1 bacteremia with pneumonia, 1 mastoiditis, 1 peritonsillar abscess, 1 pyelonephritis, 1 sinusitis, and 1 urinary tract infection (UTI).

Complete demographic data for the primary and secondary cohorts are presented in S3 Table.

### BV performance

The diagnostic accuracy of the BV score was evaluated in comparison to the reference standard diagnoses. In the primary cohort (n = 214), there were 16 true positive results, 162 true negative results, 2 false negatives and 14 false positives. The clinical details of the two false negative cases are given in S4 Table. The area under the receiver operator curve was 0.96 (95% confidence interval (CI): 0.89–1.00; S2 Fig). BV score attained sensitivity of 88.9% (74.4–100), specificity of 92.1% (88.1–96.0), PPV 53.3% (35.5–71.2) and NPV 98.8% (97.1–100). Positive likelihood ratio was 11.18 (6.59–18.97), and negative likelihood ratio was 0.12 (0.03–0.45). The rate of equivocal BV scores was 9.4%. Similar performance was observed for the secondary cohort (S5 Table).

The distribution of the BV score across the bacterial, suspected bacterial, indeterminate, suspected viral and viral reference standard diagnoses shows that high confidence BV scores (less than 10 or higher than 90) were obtained for 61.7% of the study population (S3 Fig).

### Comparison between physician’s etiological suspicion and BV score

After initial examination of the patients, physicians were asked to fill out a questionnaire giving the suspected clinical syndrome and their diagnostic suspicion (S1 Fig). Questionnaires were completed for 208 out of the 214 patients with reference standard bacterial or viral diagnoses. The physician’s diagnostic suspicion was viral for 166 cases (79.8%), bacterial for 31 cases (14.9%), mixed bacterial and viral for 1 case (0.5%), non-infectious for 1 case (0.5%), and ’I don’t know’ for 9 cases (4.3%).

**Fig 2:**
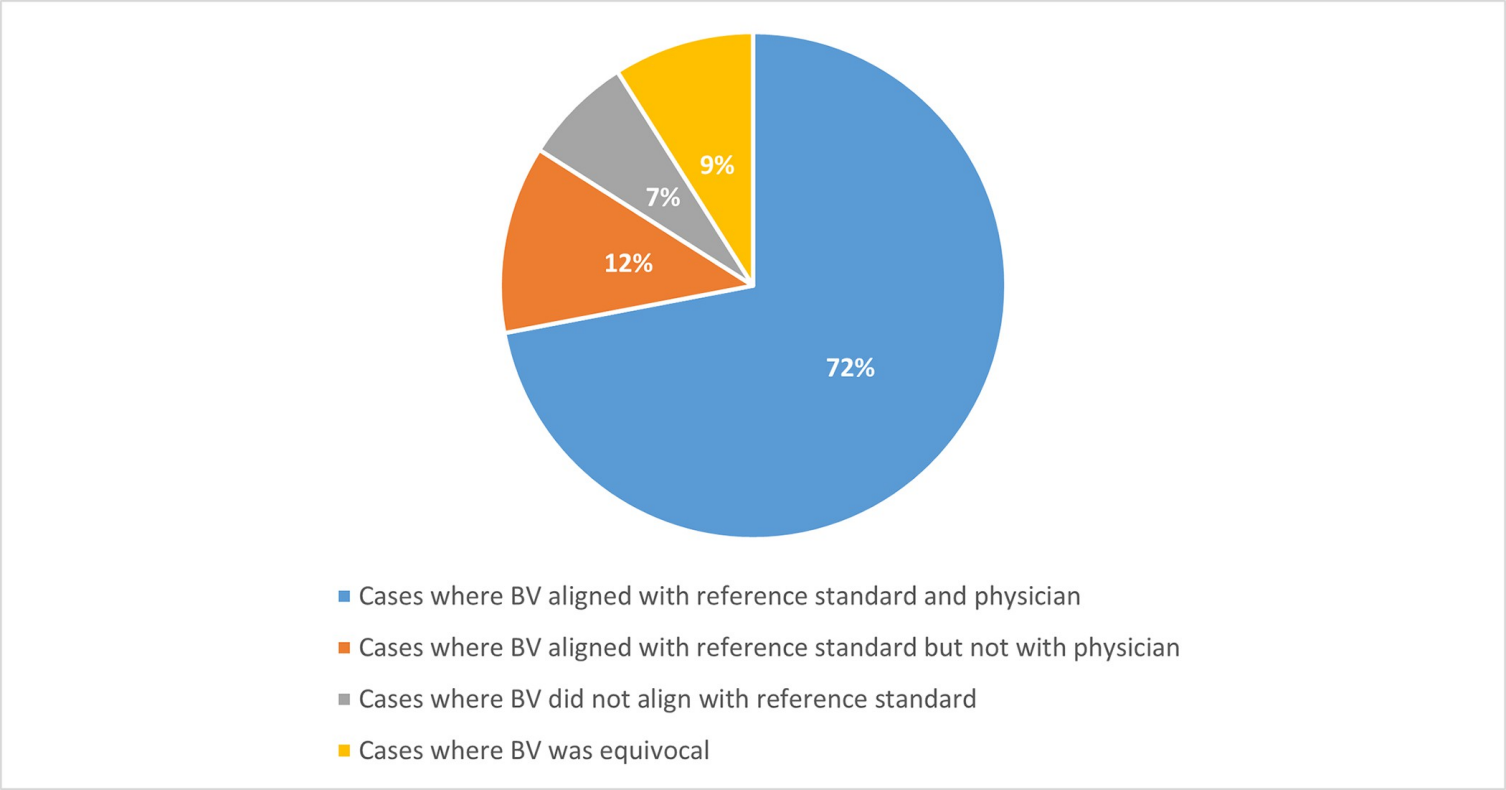
BV score versus adjudication-based reference standard diagnosis and physician label. Data are shown for the primary (reference standard bacterial/viral) cohort for the cases with completed questionnaires; n = 208.

To evaluate the potential of timely BV scores to impact the physician’s etiological suspicion, BV results were compared to the reference standard diagnosis and to the physician’s suspicion (Fig 2). BV results matched the physician’s suspicion and the reference standard diagnosis in 72% of the cases (149/208); of these, 52% (77/149) were labeled by the physician with low confidence (Viral ++, Viral +, Bacterial ++ or Bacterial +), meaning that the BV result could reinforce the physician’s suspicion. In 12% of the cases (25/208), the BV result aligned with the reference standard diagnosis but not with the physician’s suspicion, and therefore could correct the physician’s etiologic diagnosis. BV results did not align with the reference standard diagnosis in 7% of the cases (14/208) and were equivocal in 9% of the cases (20/208).

S6 Table provides clinical details for a selection of cases where the BV score could potentially correct or reinforce the physician’s initial diagnosis. For example, there were three patients with reference standard bacterial diagnoses and bacterial BV scores where the physician initially suspected a viral infection. Notably, the questionnaires were filled after initial intake, and during ED evaluation of these patients, their general condition and/or other test results prompted the physician to administer antibiotics.

**Fig 3:**
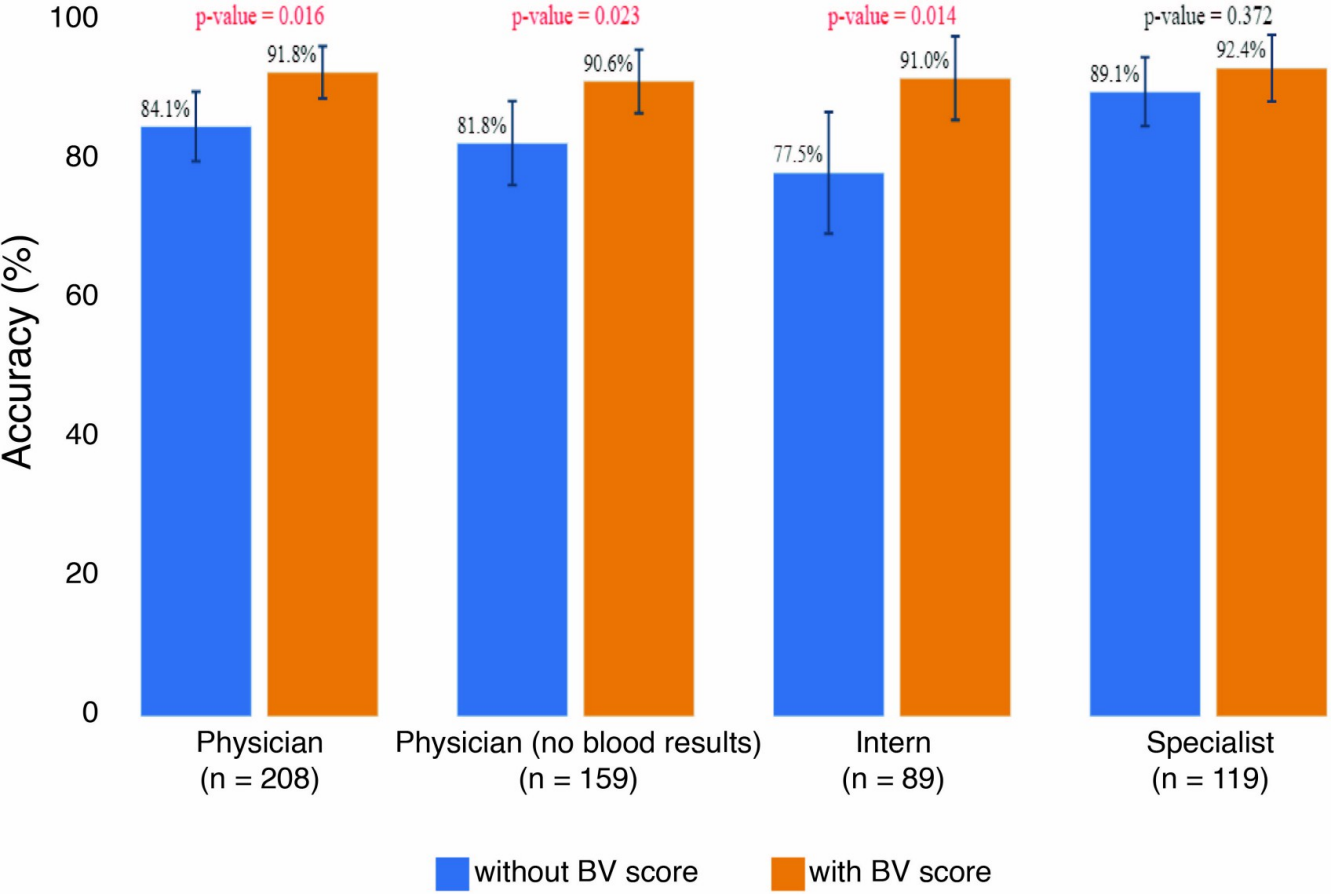
Diagnostic accuracy rate of etiological suspicion at the time of patient examination. The calculations and assumptions are explained in Methods. Error bars indicate 95% confidence intervals. P-values were computed using a one-sided Fisher-exact test; significant p-values appear in red text. Data are shown for the primary (reference standard bacterial/viral) cohort that had completed questionnaires; n = 208.

Taking the data together, the information provided by the BV score could potentially reduce diagnostic error by ~2-fold, from 15.9% to 8.2% (p = 0.016; Fig 3). The impact of the BV score on reducing diagnostic error is estimated to be greater when the physician is more junior, from 22.5% to 9% (2.5-fold; p = 0.014).

## Discussion

Fever is often the chief complaint at pediatric ED presentation, and the cause is not always apparent. After vital measurements, history taking and physical examination, the physician weighs up possible diagnoses and decides if there is need for further evaluation and treatment. In the present study we found that availability of the BV score at this point in the patient journey could potentially aid in the decision of whether to discharge or refer him/her for other diagnostic testing (e.g., chest X-ray or further bloodwork). This study found that the BV score can reinforce the physician’s suspicion and support their decision-making process, and it has potential to reduce the overall diagnostic error rate by ~2-fold.

The BV score was derived by screening a panel of 600 host proteins that may be differentially expressed in response to infection, and evaluating multiple feature selection algorithms and computational models to select the most discriminative signature [7]. In two blinded pediatric validation studies, it exhibited high performance in distinguishing between bacterial and viral infections with sensitivity, specificity and equivocal rates of 86.4% (95% confidence interval, CI: 77.4–95.4), 91.9% (95%CI: 89.2–94.6) and 12.6%, respectively, and sensitivity, specificity and equivocal rates of 93.8% (95%CI: 87.8–99.8), 89.8% (95%CI: 85.6–94.0) and 11.7%, respectively [8, 9]. The BV score demonstrated superiority to parameters and biomarkers in routine use, including CRP and procalcitonin (PCT) [7–10, 12]. This study is the first attempt to correlate between physicians’ diagnoses and the BV score, to assess its potential utility in the ED setting.

Patients were excluded from the primary objective analysis if the physician initially suspected a UTI based on symptoms or dipstick urinalysis. This is because it was assumed that in real-world practice the physician would be unlikely to use BV in febrile UTI workup. Notably, in a previous study where a sub-analysis was performed for patients with a discharge diagnosis of UTI, BV correctly yielded a bacterial score for 15 of the 16 reference standard bacterial cases [8]. Patients were also excluded if there was a suspicion of acute GE and this aligns with the current manufacturer’s limitations of use for the BV score.

It is notable that the two false negatives (cases that were bacterial according to the reference standard and yielded viral BV scores) were both mild illnesses. One case was a viral infection with a secondary localized bacterial infection, otitis media, which may not have triggered systemic immune inflammatory markers. The other case was a viral infection, with concomitant UTI. Both cases were prescribed antibiotic treatment.

A notable strength of the study is that it is the first to investigate the ED physicians’ diagnostic suspicion and compare it with the result of a new host-based test for differentiating between bacterial and viral infection. An additional strength is the rigorous reference standard for etiology, which was based on adjudication that involved extensive data collection and a panel of experienced experts blinded to one another’s etiological classification. A study limitation is the low bacterial prevalence, although it falls within the range reported in previous studies, with the proportion of bacterial infections in children with FWS and RTIs presenting at ED ranging from 0.02%–13% and 26%–28%, respectively [9]. An additional limitation is a possible bias due to including only children for whom the ED physician deemed blood tests necessary. Another limitation is that the study was conducted at a single pediatric hospital. Nevertheless, the medical site is a pediatric tertiary hospital with over 52,000 ED visits per year, and the performance results are comparable to previous studies [7, 9, 12]. A methodological limitation is that cases without reference standard diagnoses (indeterminates) were removed from the diagnostic accuracy calculations. Even after thorough evaluation of comprehensive patient data, including follow up, these cases posed diagnostic uncertainty for the adjudication experts. Future studies should explore ways to assign reference standard diagnoses also to these cases. Lastly, a limitation is that BV test results were not available to the physician, therefore this study only estimated the potential effect of BV on etiological diagnostic accuracy.

In summary, availability of the BV test when evaluating a febrile child in the ED has potential to reduce diagnostic uncertainty. Future utility studies employing a rapid measurement platform are warranted, so that the impact on actual ED practice can be assessed for a BV test result available in real-time.

## Supporting information

S1 File (XLSX)

S1 Table Standards for the reporting of diagnostic accuracy studies (STARD) checklist. (DOCX)

S2 Table The reference standard diagnosis matrix. (DOCX)

S3 Table Demographics of primary (bacterial/viral) and secondary (bacterial/viral/suspected) analysis cohorts. (DOCX)

S4 Table Bacterial cases that received a viral BV score (false negative cases). (DOCX)

S5 Table Performance of BV in secondary (bacterial/viral/suspected) analysis cohort (n = 253). (DOCX)

S6 Table Examples of cases where BV could potentially correct or reinforce physician suspicion. (DOCX)

S1 Fig Physician label questionnaire. This form was filled by the ED attending physician after obtaining medical history and physical examination. It documents the extent of available clinical information at the time of questionnaire completion (e.g., laboratory tests, urinalysis). Physician’s suspicion of clinical syndrome, initial etiologic diagnosis and degree of diagnostic confidence were also documented.
(DOCX)

S2 Fig BV score performance. The area under the receiver operating characteristic curve analysis of diagnostic performance is shown for the primary (reference standard bacterial/viral) cohort; n = 214.
(DOCX)

S3 Fig Distribution of BV scores according to reference standard diagnosis. Each circle represents a patient in the study population (n = 287). Red line corresponds to group median and red circle corresponds to group average.
(DOCX)
