# Supplementary material for: Bacterial vs viral etiology of fever: A prospective study of a host score for supporting etiologic accuracy of emergency department physicians
Source: PLoS One. 2023 Jan 30;18(1):e0281018. doi: 10.1371/journal.pone.0281018 (PMC9886241; doi:10.1371/journal.pone.0281018)
Supplement: S2 Table — (DOCX) [file pone.0281018.s003.docx]

## **S2** Table. The reference standard diagnosis matrix

| Reference standard diagnosis | Required adjudication classification |
| --- | --- |
| Bacterial infection | All experts adjudicate bacterial classification |
| Suspected bacterial infection | The majority but not all experts adjudicate bacterial classification |
| Indeterminate | No majority among experts or majority of experts adjudicate indeterminate |
| Suspected viral infection | The majority but not all experts adjudicate viral classification |
| Viral infection | All experts adjudicate viral classification |
