# Supplementary material for: Bacterial vs viral etiology of fever: A prospective study of a host score for supporting etiologic accuracy of emergency department physicians
Source: PLoS One. 2023 Jan 30;18(1):e0281018. doi: 10.1371/journal.pone.0281018 (PMC9886241; doi:10.1371/journal.pone.0281018)
Supplement: S3 Table — (DOCX) [file pone.0281018.s004.docx]

## **S3 Table.** Demographics of primary (bacterial/viral) and secondary (bacterial/viral/suspected) analysis cohorts

**
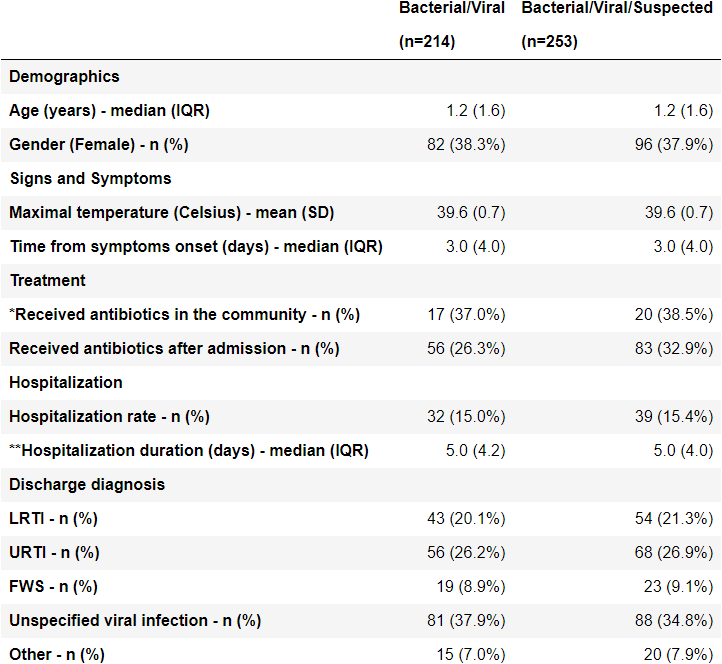
** Indeterminate cases were excluded.

* Among patients with available information (n = 46, 52 respectively).

** Among hospitalized patients (n = 32, 39 respectively).
