# Supplementary material for: Bacterial vs viral etiology of fever: A prospective study of a host score for supporting etiologic accuracy of emergency department physicians
Source: PLoS One. 2023 Jan 30;18(1):e0281018. doi: 10.1371/journal.pone.0281018 (PMC9886241; doi:10.1371/journal.pone.0281018)
Supplement: S4 Table — (DOCX) [file pone.0281018.s005.docx]

## **S4 Table.** Bacterial cases that received a viral BV score (false negative cases)

| **Patient #4611** | **BV score** | **Adjudication label** | **Physician label** | **Gender** | **Age (m)** | **Discharge diagnosis** | **Max temp** | **Hospital admission** | **Abx** | **Microbiology** |
| --- | --- | --- | --- | --- | --- | --- | --- | --- | --- | --- |
|  | 30 | Bacterial | Viral ++ | Female | 3 | UTI | 39.6 | Yes | Yes | Rhinovirus (PCR).  E. Coli, CFU/ml >100,000 (Urine culture) |
|  | **Clinical details** | | | | | | | | | |
|  | Presented with 3-day history of fever. Physical exam described a well appearing, vital patient with fever and rhinorrhea with no other significant findings. Main lab results- WBC 15.9K, ANC 8.3K, CRP 35 mg/L. Urinalysis was positive for leukocytes. | | | | | | | | | |
| **Patient #4922** | **BV score** | **Adjudication label** | **Physician label** | **Gender** | **Age (m)** | **Discharge diagnosis** | **Max temp** | **Hospital admission** | **Abx** | **Microbiology** |
|  | 6 | Bacterial | Viral +++ | Female | 12 | AOM | 39.8 | No | Yes | Adenovirus and Parainfluenza virus 3 (PCR).  Strep. Pneumoniae, susceptible to all antibiotics  (Ear culture) |
|  | **Clinical details** | | | | | | | | | |
|  | Presented with 5-day history of fever along with cough and rhinorrhea. Initial physical examination revealed redness in throat with normal otoscopy and no other findings. Revision of the otoscopy revealed bilateral otitis media with effusion. | | | | | | | | | |
