# Supplementary material for: Bacterial vs viral etiology of fever: A prospective study of a host score for supporting etiologic accuracy of emergency department physicians
Source: PLoS One. 2023 Jan 30;18(1):e0281018. doi: 10.1371/journal.pone.0281018 (PMC9886241; doi:10.1371/journal.pone.0281018)
Supplement: S5 Table — (DOCX) [file pone.0281018.s006.docx]

## **S5** Table. Performance of BV in secondary (bacterial/viral/suspected) analysis cohort (n = 253)

|  | Statistic | CI (95%) |
| --- | --- | --- |
| AUC | 0.91 | 0.84-0.99 |
| Bacterial prevalence (%) | 11.1 |  |
| Sensitivity (%) | 76.9 | 60.7-93.1 |
| Specificity (%) | 90.6 | 86.5-94.6 |
| PPV (%) | 51.3 | 35.6-67.0 |
| NPV (%) | 96.8 | 94.3-99.2 |
| LR+ | 8.14 | 5.05-13.11 |
| LR- | 0.25 | 0.13-0.51 |
| Equivocal (%) | 10.3 |  |

AUC, area under the receiver operating characteristic curve; PPV, positive predictive value; NPV, negative predictive value; LR+, positive likelihood ratio; LR-, negative likelihood ratio.
