# Supplementary material for: Bacterial vs viral etiology of fever: A prospective study of a host score for supporting etiologic accuracy of emergency department physicians
Source: PLoS One. 2023 Jan 30;18(1):e0281018. doi: 10.1371/journal.pone.0281018 (PMC9886241; doi:10.1371/journal.pone.0281018)
Supplement: S6 Table — (DOCX) [file pone.0281018.s007.docx]

## **S6 Table.** Examples of cases where BV could potentially correct or reinforce physician suspicion

| Patient #4609 | BV score | Expert label | Physician label | Gender | Age (m) | Discharge diagnosis | Max temp. | Hospital admit | Abx | Microbiology |
| --- | --- | --- | --- | --- | --- | --- | --- | --- | --- | --- |
|  | 16 | V | I don’t know | F | 30 | Pneumonia due to RSV | 40.2 | Yes | Amoxicillin | Negative blood culture; RSV A and rhinovirus  (PCR) |
|  | Clinical details | | | | | | | | | |
|  | Presented with 2-day history of fever, shortness of breath, cough, wheezing and rhinorrhea. Physical exam showed fever with tachypnea, tachycardia, chest auscultation revealed LLL wheezes. Main lab results- WBC 5.4K, ANC 3.5K, CRP 43.4 mg/L. Chest radiography revealed bilateral infiltrates. She was managed with hypertonic saline inhalations and amoxicillin PO. | | | | | | | | | |
| Patient #4601 | BV score | Expert label | Physician label | Gender | Age (m) | Discharge diagnosis | Max temp. | Hospital admit | Abx | Microbiology |
|  | 4 | V | I don’t know | M | 11 | Acute bronchiolitis | 39.2 | Yes | Cefuroxime | RSV B, adenovirus and rhinovirus  (PCR). |
|  | Clinical details | | | | | | | | | |
|  | One day prior to PED presentation he developed shortness of breath with fever, post-tussive emesis, decreased appetite and low urine output. Of note, medical history of hyper-reactive airway disease. Physical exam showed fever with tachypnea, tachycardia, lung findings consistent with bronchiolitis and oxygen saturation of 88% on room air. Main lab results- WBC 15.45K, ANC 6.8K, CRP 158 mg/L. Chest radiography revealed bilateral hyperinflation and infiltrates. He was managed with supplemental oxygen, corticosteroids, inhalations, parenteral cefuroxime. Given his ongoing dyspnea, he was given adrenalin inhalations. | | | | | | | | | |
| Patient #4562 | BV score | Expert label | Physician label | Gender | Age (m) | Discharge diagnosis | Max temp. | Hospital admit | Abx | Microbiology |
|  | 16 | V | B+ | F | 11 | Occult bacteremia not ruled out | 40.4 | No | Ceftriaxone | Negative blood and urine culture; Adenovirus (PCR). |
|  | Clinical details | | | | | | | | | |
|  | Presented with 2-day history of fever, excessive drooling and loss of appetite. Of note, medical history of preterm birth (34 weeks gestational age), recurrent UTI. Erythematous tonsils with bilateral exudate on physical exam. Main lab results- WBC 16.5K, ANC 9.7K, CRP 91.9 mg/L. Negative dipstick urinalysis. | | | | | | | | | |
| Patient #4620 | BV score | Expert label | Physician label | Gender | Age (m) | Discharge diagnosis | Max temp. | Hospital admit | Abx | Microbiology |
|  | 97 | B | V+++ | M | 13 | Pneumonia | 39.2 | Yes | Ceftriaxone | Negative blood culture; Influenza H1N1 (PCR). |
|  | Clinical details | | | | | | | | | |
|  | Presented with 7-day history of fever, shortness of breath, cough and rhinorrhea. Physical exam showed bilateral eye discharge. Main lab results- WBC 23.9K, ANC 16.7K, CRP 113 mg/L. Chest radiography revealed LLL infiltrate with a small pleural effusion. He was managed with one dose of Ceftriaxone at the PED and Cefuroxime on admission. He was discharged with Amoxicillin. | | | | | | | | | |
| Patient #4659 | BV score | Expert label | Physician label | Gender | Age (m) | Discharge diagnosis | Max temp. | Hospital admit | Abx | Microbiology |
|  | 78 | B | V++ | F | 22 | Pneumonia | 39.5 | No | Ceftriaxone | Negative blood culture; No virus detected  (PCR). |
|  | Clinical details | | | | | | | | | |
|  | Presented with 6-day history of fever, productive cough and decreased appetite. Physical exam showed rhinorrhea and chest auscultations revealed bilateral minimal rales. Main lab results- WBC 10.43K, ANC 4K, CRP 53.8 mg/L. Chest radiography demonstrated LLL alveolar infiltrate. She was managed with one dose of Ceftriaxone at the PED and discharged home with Amoxicillin. | | | | | | | | | |

B = bacterial; V = viral
