# Supplementary material for: Bacterial vs viral etiology of fever: A prospective study of a host score for supporting etiologic accuracy of emergency department physicians
Source: PLoS One. 2023 Jan 30;18(1):e0281018. doi: 10.1371/journal.pone.0281018 (PMC9886241; doi:10.1371/journal.pone.0281018)
Supplement: S1 Fig — This form was filled by the ED attending physician after obtaining medical history and physical examination. It documents the extent of available clinical information at the time of questionnaire completion (e.g., laboratory tests, urinalysis). Physician’s suspicion of clinical syndrome, initial etiologic diagnosis and degree of diagnostic confidence were also documented. (DOCX) [file pone.0281018.s008.docx]

## S1 Fig. Physician label questionnaire


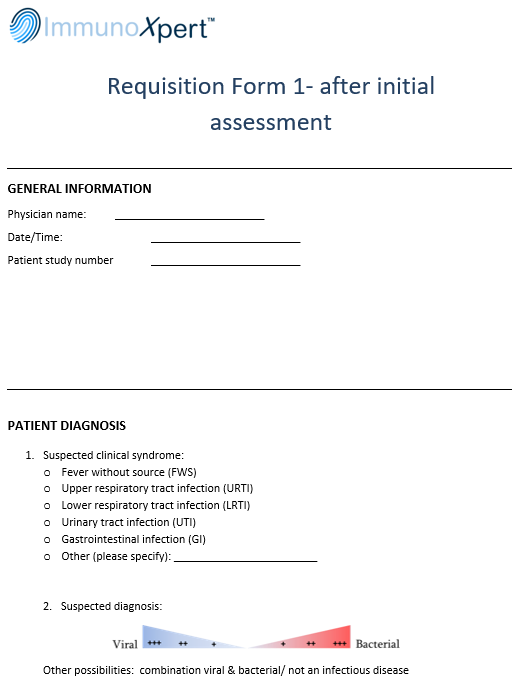


This form was filled by the ED attending physician after obtaining medical history and physical examination. It documents the extent of available clinical information at the time of questionnaire completion (e.g., laboratory tests, urinalysis). Physician's suspicion of clinical syndrome, initial etiologic diagnosis and degree of diagnostic confidence were also documented.
