# Supplementary material for: Bacterial vs viral etiology of fever: A prospective study of a host score for supporting etiologic accuracy of emergency department physicians
Source: PLoS One. 2023 Jan 30;18(1):e0281018. doi: 10.1371/journal.pone.0281018 (PMC9886241; doi:10.1371/journal.pone.0281018)
Supplement: S2 Fig — The area under the receiver operating characteristic curve analysis of diagnostic performance is shown for the primary (reference standard bacterial/viral) cohort; n = 214. (DOCX) [file pone.0281018.s009.docx]

## S2 Fig. BV score performance


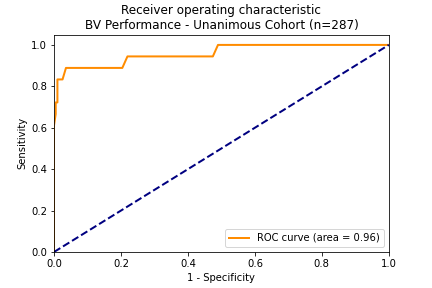


The area under the receiver operating characteristic curve (AUROC) analysis of diagnostic performance is applied to the entire cohort. Data is shown for the primary (reference standard bacterial/viral) cohort; n=214.
