# Supplementary material for: Bacterial vs viral etiology of fever: A prospective study of a host score for supporting etiologic accuracy of emergency department physicians
Source: PLoS One. 2023 Jan 30;18(1):e0281018. doi: 10.1371/journal.pone.0281018 (PMC9886241; doi:10.1371/journal.pone.0281018)
Supplement: S3 Fig — Each circle represents a patient in the study population (n = 287). Red line corresponds to group median and red circle corresponds to group average. (DOCX) [file pone.0281018.s010.docx]

## S3 Fig. Distribution of BV scores according to reference standard diagnosis


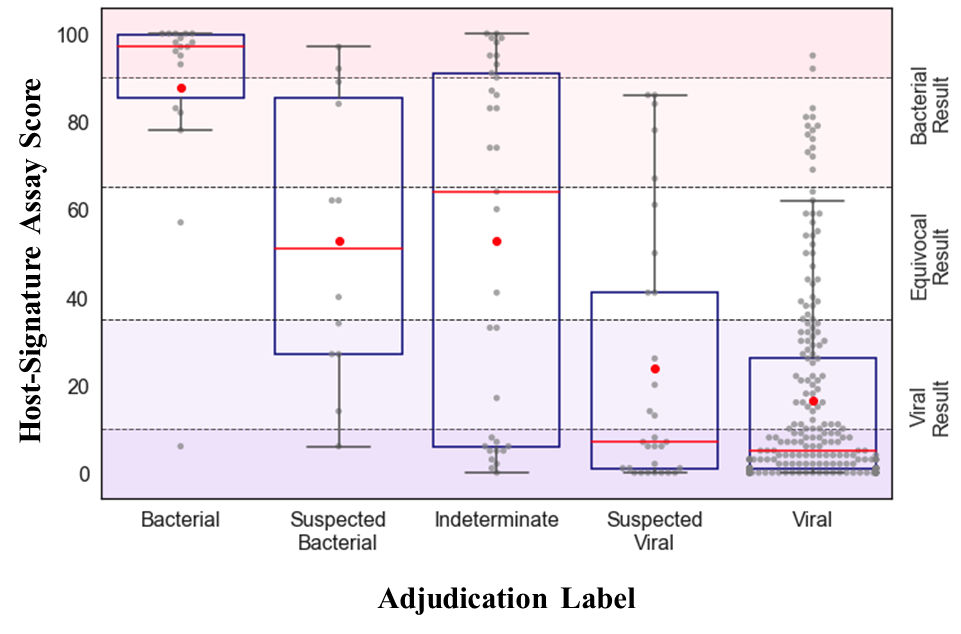

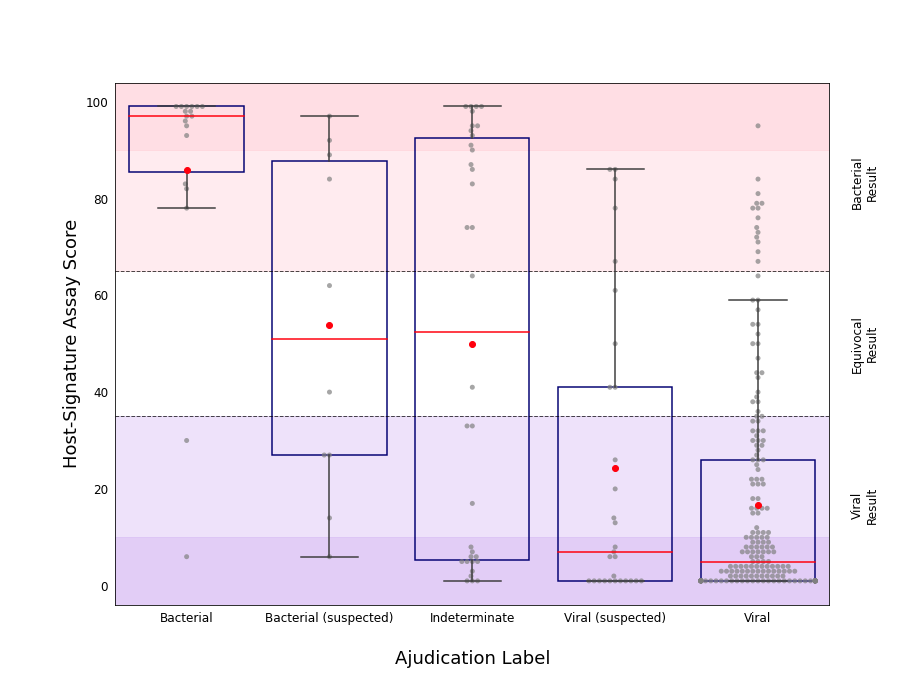


**BV Score**

Each circle represents a patient in the study population (n = 287). Red line corresponds to group median and red circle corresponds to group average.

Reference standard diagnosis
